# The high-quality sequencing of the *Brassica rapa* ‘XiangQingCai’ genome and exploration of genome evolution and genes related to volatile aroma

**DOI:** 10.1093/hr/uhad187

**Published:** 2023-09-15

**Authors:** Zhaokun Liu, Yanhong Fu, Huan Wang, Yanping Zhang, Jianjun Han, Yingying Wang, Shaoqin Shen, Chunjin Li, Mingmin Jiang, Xuemei Yang, Xiaoming Song

**Affiliations:** Suzhou Academy of Agricultural Sciences, Suzhou, Jiangsu 215155, China; College of Life Sciences, North China University of Science and Technology, Tangshan, Hebei 063210, China; Suzhou Academy of Agricultural Sciences, Suzhou, Jiangsu 215155, China; Suzhou Polytechnic Institute of Agriculture, Suzhou, Jiangsu 215008, China; Suzhou Academy of Agricultural Sciences, Suzhou, Jiangsu 215155, China; Suzhou Academy of Agricultural Sciences, Suzhou, Jiangsu 215155, China; College of Life Sciences, North China University of Science and Technology, Tangshan, Hebei 063210, China; College of Life Sciences, North China University of Science and Technology, Tangshan, Hebei 063210, China; Suzhou Academy of Agricultural Sciences, Suzhou, Jiangsu 215155, China; Suzhou Academy of Agricultural Sciences, Suzhou, Jiangsu 215155, China; College of Life Sciences, North China University of Science and Technology, Tangshan, Hebei 063210, China

## Abstract

‘Vanilla’ (XQC, *brassica variety chinensis*) is an important vegetable crop in the Brassica family, named for its strong volatile fragrance. In this study, we report the high-quality chromosome-level genome sequence of XQC. The assembled genome length was determined as 466.11 Mb, with an N50 scaffold of 46.20 Mb. A total of 59.50% repetitive sequences were detected in the XQC genome, including 47 570 genes. Among all examined Brassicaceae species, XQC had the closest relationship with *B. rapa* QGC (‘QingGengCai’) and *B. rapa* Pakchoi. Two whole-genome duplication (WGD) events and one recent whole-genome triplication (WGT) event occurred in the XQC genome in addition to an ancient WGT event. The recent WGT was observed to occur during 21.59–24.40 Mya (after evolution rate corrections). Our findings indicate that XQC experienced gene losses and chromosome rearrangements during the genome evolution of XQC. The results of the integrated genomic and transcriptomic analyses revealed critical genes involved in the terpenoid biosynthesis pathway and terpene synthase (TPS) family genes. In summary, we determined a chromosome-level genome of *B. rapa* XQC and identified the key candidate genes involved in volatile fragrance synthesis. This work can act as a basis for the comparative and functional genomic analysis and molecular breeding of *B. rapa* in the future.

## Introduction

‘XiangQingCai’ (XQC, *Brassica rapa* ssp*. chinensis*) belongs to the genus *Brassica* of the Brassicaceae family, along with *Arabidopsis thaliana*. *Brassica* includes numerous vegetables and oilseed plants [1]. The *Brassica* U triangle model describes the genomic relationships of three diploid and three allopolyploid *Brassica* species, namely, *Brassica oleracea* (CC genome), *B. rapa* (AA genome), *Brassica nigra* (BB genome), *Brassica carinata* (BBCC genome), *Brassica juncea* (AABB genome), and *Brassica napus* (AACC genome) [1, 2].

At present, the genomes of the six species from the *Brassica* U triangle model have been completed. In particular, several studies have released these genomes, including *B. rapa* [3–11], *B. oleracea* [12–17], *B. nigra* [18], *B. napus* [19–26], *B. carinata* [1, 27], and *B. juncea* [28, 29]*.* All of the six *Brassica* species underwent a recent whole-genome triplication (WGT) event since their divergence from *A. thaliana* [1]. These genomes provide rich resources for genomic research on *Brassica* species [30, 31].

*B. rapa* has a very rich morphological and genetic diversity, and contains a diverse group of subspecies, such as pekinensis, chinensis, trilocularis, oleifera, parachinensis, rapa, narinosa, and nipposinica [32, 33]. As an important member of the U triangle, several genomes of the *B. rapa* subspecies have been released successively. These genomes provide rich data resources for the study of the genomic microevolution of *B. rapa*. As a chinensis subspecies of *B. rapa*, XQC has a strong fragrance, yet its genome remains to be deciphered.

Here, the genome of XQC was sequenced using several methods, including PacBio HiFi, Illumina, and Hi-C. The XQC genome provides a distinctive tool to explore the mechanisms that contribute to the regulatory pathway of fragrance synthesis. In addition to assisting in the molecular breeding of the fragrance of *Brassica*, the XQC genome sequences can also contribute to the future genomics analysis of Brassicaceae.

**Table 1:** Sequencing, assembly and annotation statistics of the XQC genome

Genomic feature	Values
Total genome sequencing data	150.73 Gb
Total genome sequencing coverage	329.20 X
Genome size (estimated)	457.87 Mb
Genome size (assembled)	466.11 Mb
Contig N50 of the assembly	15.15 Mb
Scaffold N50 of the assembly	46.20 Mb
Mapping rate	99.75%
BUSCO	99.40%
CEGMA	97.58%
Repeat sequences of genome	59.50%
Number of protein-coding genes	47 570
Functional annotated genes	47 366
Number of noncoding RNAs	60535

**Figure 1:**
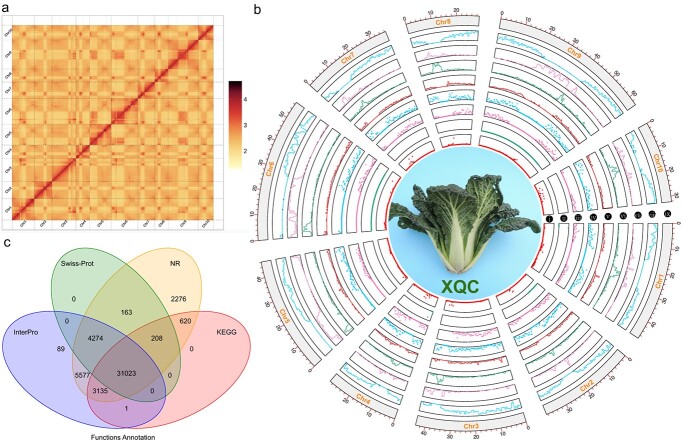
Hi-C distribution map, genome features, and gene annotation of the XQC genome. (**a**) Interactions of the ten XQC chromosomes using Hi-C technology. (**b**) Distribution of gene density and different repeat sequences of the ten XQC chromosomes (window size of 200 Kb). i–viii show the TRF, SSRs, SINE, LINE, DNA repeats, Copia, Gypsy, and gene distribution, respectively. ix shows the ten XQC chromosomes. The inner circle is the morphology of the XQC plant. (**c**) Venn diagram of gene functional annotation for XQC using four databases.

## Results

### XQC genome sequencing, assembly, and assessment

The *B. rapa* XQC genome was sequenced by the PacBio HiFi (Pacific Biosciences, San Diego, CA, USA) and Illumina sequencing technologies (Illumina, San Diego, CA, USA) (Table S1, see online supplementary material). Based on the K-mer method, the estimated heterozygosity rate of the XQC genome was approximately 0.23% and estimated genome size was 457.87 Mb (Table 1; Tables S2 and S3, Fig. S1, see online supplementary material). A total of 51.13 Gb of data, with an average of 111.67×, was obtained using the Illumina sequencer. PacBio HiFi was used to obtain 28.25 Gb of data (61.70×) (Tables S1 and S4, see online supplementary material). Furthermore, 71.35 Gb (155.83×) of Hi-C sequencing data was generated to assist the assembly of the XQC genome (Fig. 1a; Table S1, see online supplementary material). In total, 150.73 Gb (329.20×) of genomic data was obtained to perform the XQC genome assembly. The assembled XQC genome was approximately 466.10 Mb (contig N50 of 15.15 Mb and scaffold N50 of 46.20 Mb), indicating its high quality (Table 1; Table S5, see online supplementary material). In total, 431.02 Mb sequences (92.47%) were mapped to 10 XQC chromosomes (Fig. 1b; Table S6, see online supplementary material).

The read mapping rate exceeded 99.75%, demonstrating the relative completeness of the assembled XQC genome (Table S7, see online supplementary material). Furthermore, the CEGMA and BUSCO assessments also demonstrated the high quality of the XQC genome. A total of 98.39% (244) of core genes was identified in the XQC genome by the CEGMA analysis (Table S8, see online supplementary material). Similarly, 99.4% of 1614 genes were detected in the XQC genome using the BUSCO assessment (Table S9, see online supplementary material).

### Genome annotation

We detected 59.50% of the estimated XQC genomes to be repetitive sequences (Fig. 1c; Table S10, see online supplementary material). A total of 218.16 Mb (43.84%) repetitive sequences were long-terminal repeats (LTRs), followed by DNA transposons (11.83%). The majority of genes and DNA transposable elements were detected in the chromosomal terminal regions, which was inversely distributed with the LTR repeat sequences on the chromosomes (Fig. 1c).

A total of 47 570 genes were predicted from the XQC genome (Figs S2 and S3, Tables S11 and S12, see online supplementary material), with 47 366 (99.57%) genes annotated using the InterPro, Swiss-Prot, NR, and KEGG databases, and 31 023 genes annotated with all four databases (Table S13, see online supplementary material). A total of 548 miRNAs, 12 371 tRNAs, 22 485 rRNAs, and 1323 snRNAs were identified in the XQC genome (Table S14, see online supplementary material).

### Gene family analysis, divergence time estimation, and positive selection analysis

**Figure 2:**
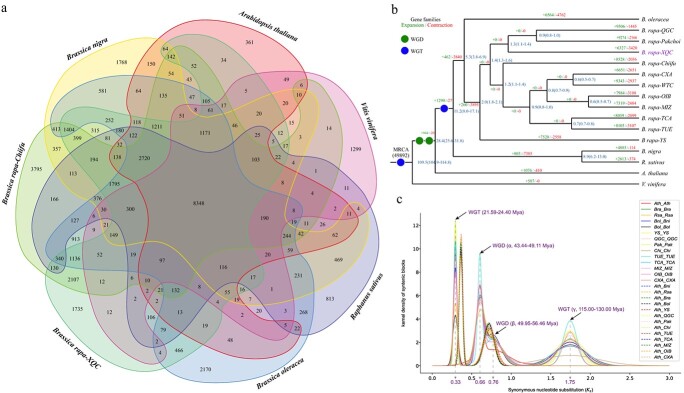
Gene family and phylogenetic relationship analysis. (**a**) Common and species-specific gene families in XQC and other six species. (**b**) Expansion/contraction of gene family and estimation of divergence time. Blue numbers indicate the divergence time on the nodes, with the confidence range in parentheses. (**c**) Ks density of syntenic genes between XQC and other examined species. Distribution curve lines were fitted with the corrected Ks values. The numbers in parentheses denote the inferred times of the related WGD and WGT events.

We detected the gene families in XQC, as well as 12 other *Brassica* species, *Raphanus sativus*, *Vitis vinifera*, and *A. thaliana* (Fig. S4, Table S15, see online supplementary material). A total of 49 906 gene families were detected in XQC and the other 15 examined species (Table S16, see online supplementary material), with 26 379 gene families containing 43 657 genes predicted in the XQC genome (Table S16, see online supplementary material). In total, 7606 common gene families and 2028 single-copy gene families were detected in 16 plants. Furthermore, specific and common gene families among the XQC and other six species were identified (Fig. 2a). A total of 1735 specific gene families were detected in XQC, exceeding the numbers in *A. thaliana* (361), *V. vinifera* (1299), and *R. sativus* (813), and lower than that of *B. nigra* (1768), *B. oleracea* (2170), and *B. rapa* Chiifu (3795) (Fig. 2a).

We identified 6327 expanded gene families in the XQC genome, which was less than that of *B. rapa* QGC (9506) and *B. rapa* Pakchoi (9274) (Fig. 2b). However, 3420 contracted gene families were predicted in the XQC genome, exceeding the number of the corresponding gene families predicted for the other species. Phylogenetic relationship and divergence time analyses were performed with the 2028 single-copy gene families of the 16 species (Fig. 2b). XQC presented the closest relationship and synteny with *B. rapa* QGC and *B. rapa* Pakchoi, exhibiting a divergence from these two species during 1.1–1.4 Mya (Fig. 2b).

Moreover, we detected 1297 positive selection genes by comparing XQC with other *B. rapa* varieties (*q*-value <0.05) (Table S17, see online supplementary material). The enrichment analysis showed that 22 terms were significantly enriched in XQC (Table S18, see online supplementary material). The first three enriched terms were spliceosome, ubiquinone and other terpenoid-quinone biosynthesis, and RNA transport, which may be involved in regulating the volatile aroma formation of XQC.

### Polyploidization and evolution of the XQC genome

**Figure 3:**
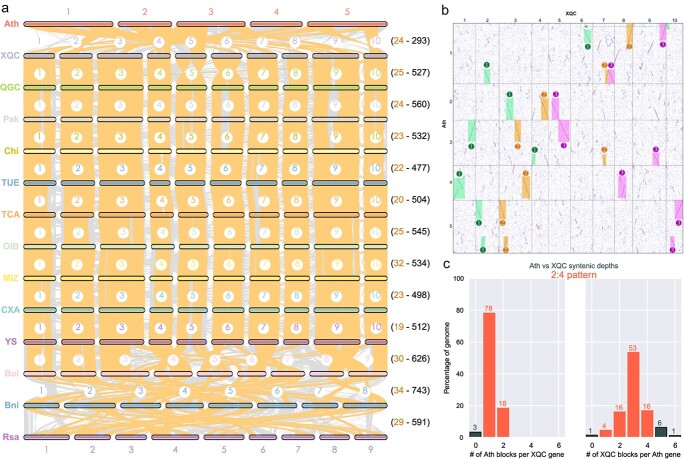
Syntenic analysis and dot plot between XQC and other related species. (**a**) Syntenic plots between XQC and other examined species. Syntenic blocks and large syntenic blocks (>500 gene pairs) are marked using gray and orange lines, respectively. The numbers in parentheses represent large syntenic blocks (>500 gene pairs) and all syntenic block numbers between two related species, respectively. The full name of the species can be found in Table S16 (see online supplementary material). (**b**) Dot-plot between XQC and *Arabidopsis thaliana* chromosomes. Three collinear regions are illustrated using rectangles labelled as 1, 2, and 3 as an example. (**c**) Depth analysis of syntenic blocks between XQC and *A. thaliana*.

The genomic evolution of XQC was explored based on the rate of the Ks values within the collinear blocks between XQC, *A. thaliana*, *R. sativus*, and 11 other *Brassica* species (Figs. 2c and 3; Fig. S5, see online supplementary material). The XQC genome exhibited four peaks in the Ks density plot (Figs. 2c; Fig. S5, see online supplementary material). This indicates the occurrence of four polyploidization events in the XQC genome, including the ancient WGT (γ) event that occurred in grape and eudicots [35], two WGD (β and α) events shared with *A. thaliana* and other Brassicaceae, and the recent WGT event shared with *R. sativus* and other *Brassica* species. We verified the occurrence of the recent WGT event in the XQC genome by combining syntenic analysis and dot plots of *A. thaliana* (Fig. 3a–b). The latest WGT event of the XQC genome occurred during 21.59–24.40 Mya (Ks = 0.33) according to the Ks density plot following the evolution rate correction (Fig. 2c; Table S19, see online supplementary material).

### Syntenic and global alignment analysis of the XQC genome

The XQC gene sequences were mapped to the *A. thaliana* genome to deduce their inter-genomic collinearity. This was then expanded to other Brassicaceae species (Fig. 3a; Figs S6–S13, see online supplementary material). We identified 293 syntenic blocks between XQC and *A. thaliana*, which was less than the number of collinear blocks (477–743) for the other Brassicaceae*.* Among these species, the highest number of collinear blocks was observed between *B. oleracea* and *B. nigra* (743). In addition, the largest number of large syntenic blocks (gene pairs >500) were detected between *B. oleracea* and *B. nigra* (34), while the lowest number of large syntenic blocks were detected between *B. rapa* CXA and *B. rapa* YS (19). Syntenic analysis also identified the greatest number of chromosome rearrangements to occur between *B. rapa* and other Brassicaceae species, as well as a strong collinear relationship between different *B. rapa* varieties (Fig. 3a; Figs S6–S13).

The dot plot revealed a 3:1 ratio of several syntenic blocks between XQC and *A. thaliana*, which also verifies the recent WGT event in the XQC genome after its divergence from *A. thaliana*. For example, the end of chromosome 3 in *A. thaliana* was syntenic with XQC chromosomes 4, 7, and 9 (Fig. 3a and b). Furthermore, we calculated the syntenic depth ratio of XQC and other examined species at the genome-wide level. The syntenic depth between XQC and other *Brassica* species and *R. sativus* was 3:3, indicating that they shared the recent WGT event after diverging from *A. thaliana*. However, the syntenic depth between *A. thaliana* and XQC was 2:4 rather than 1:3 (Fig. 3c). This indicates that the genes of the XQC genome underwent a partial loss after the recent WGT event. This phenomenon was further verified by the syntenic depth of XQC and grape, which was 6:1 rather than 12:1.

**Figure 4:**
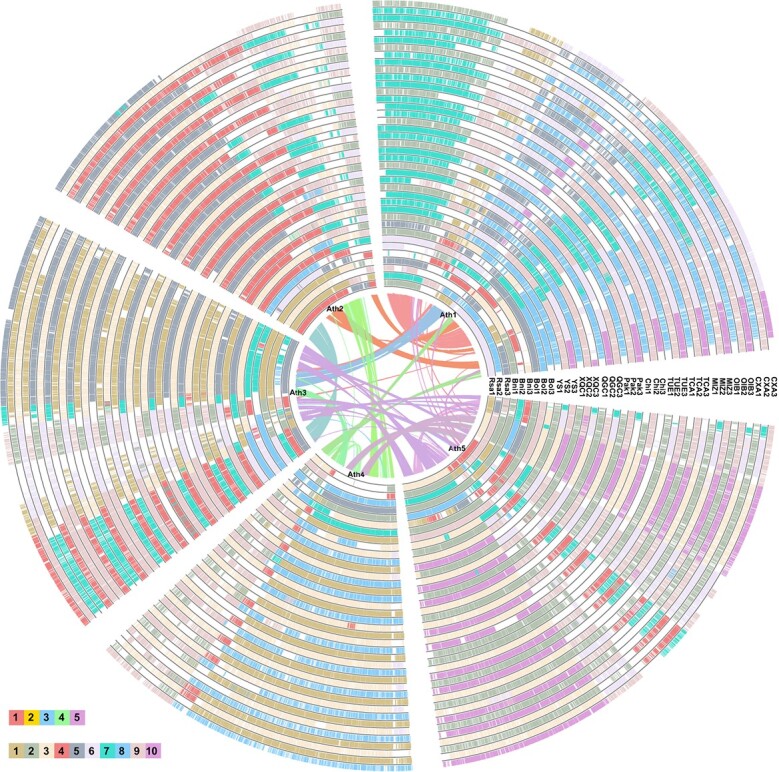
Global alignment analysis of the XQC genome and other related species. Global alignment analysis of XQC and 12 other plants with *Arabidopsis thaliana* as a reference. XQC and the 12 other genomes were divided into three subgenomes. Syntenic genes between *A. thaliana* and each subgenome of the examined species are presented in the circle plot. The curves of the inner circle plot depict the collinear blocks between five *A. thaliana* chromosomes.

We performed a global alignment of XQC, 12 other *Brassica* species, and *R. sativus* using *A. thaliana* as a reference to explore the gene loss and retention in these genomes (Fig. 4; Table S20, see online supplementary material). Each genome of XQC and the 12 other *Brassica* genomes were further classified into three subgroups based on the recent WGT event. The results clearly revealed the loss and retention of the collinear genes in the three sub-genomes of these *Brassica* species. For example, among the three sub-genomes of XQC, 8843, 8248, and 9310 collinear genes were identified to refer to the *A. thaliana* genome (Fig. 4,;Table S20, see online supplementary material). This is the first time that the XQC, 12 *Brassica* species, and *R. sativus* genomes were jointly compared based on the genome of *A. thaliana*. The findings provide a rich resource for future research on the evolution and function of collinear genes between these genomes.

### Exploration of genes involved in volatile aroma

#### Terpenoid biosynthesis genes

**Figure 5:**
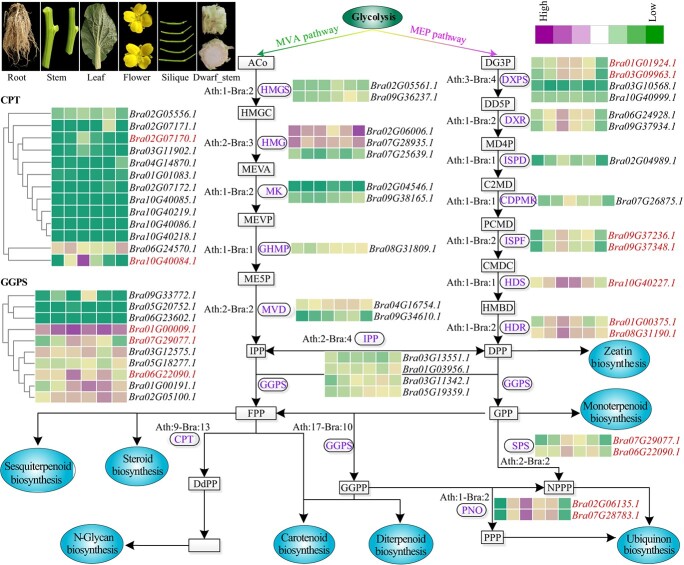
Overview of the terpenoid biosynthesis pathway in XQC. Two synthesized terpene precursor pathways, namely, the methylerythritol phosphate (MEP) and mevalonic acid (MVA) pathways. The notation ‘N-N’ indicates that the N homologous genes were identified in the *Arabidopsis thaliana* and XQC genomes. The expression of each gene was identified in the six different tissues of XQC. The purple and green colors showed high and low expression levels in the related tissues, respectively. Eight sub-biosynthesis pathways were marked with blue ovals. Table S22 reports the compound abbreviations and Table S23 indicates the gene expression values (see online supplementary material). Gene names in red indicate genes with an up-regulated expression in leaf compared to other tissues.

The genes involved in the terpenoid biosynthesis pathway were used for the formation of plant imparted volatile aroma. We identified 47 Arabidopsis genes from the terpenoid biosynthesis pathway. Using these genes as seeds, we predicted the homologous genes in XQC using BLASTP and the Pfam domains (Tables S21–S22; see online supplementary material). In the regulatory pathway, multiple nodes in XQC exhibited the same gene number as *A. thaliana* (Fig. 5). However, the majority of nodes have more than one gene copies in XQC due to the specific WGT event; for example the DXR, MK, HMGS, IPP, and CPT genes (Fig. 5; Table S21, see online supplementary material). Interestingly, the number of GGPS in XQC (10) was less than that in *A. thaliana* (17), indicating that some GGPS genes were lost in XQC after diverging from *A. thaliana*.

The leaves of XQC have a strong volatile aroma, and thus we identified significantly up-regulated terpenoid biosynthesis genes in leaf rather than in other tissues, providing candidate gene resources for flavor research. Finally, we identified 16 up-regulated genes in leaf, including 2 *DXPS*, 2 *ISPF*, 1 *HDS*, 2 *HDR*, 2 *SPS*, 2 *PNO*, 2 *CPT*, and 3 *GGPS* (Fig. 5). The aforementioned up-regulated gene *Bra07G28783.1* (*PNO*, FPMK: 238.29) showed the highest expression value in leaf, followed by *Bra02G06135.1* (*PNO*, FPMK: 189.28) and *Bra10G40227.1* (*HDS*, FPMK: 166.38) (Table S23, see online supplementary material). We speculated that these genes played critical roles in the volatile aroma synthesis of XQC leaves.

#### Terpene synthase gene family

We detected terpene synthase (TPS) family genes in XQC and *A. thaliana* due to its importance in terpenoid biosynthesis. Among the 34 TPS genes detected in XQC, only 23 (67.65%) TPS genes exhibited two conserved domains. Furthermore, eight of these genes only contained the domain PF03936, while three genes only contained the domain PF01397. In *A. thaliana*, 33 of the 34 (97.06%) TPS genes had two domains, while the remaining gene only contained the domain PF03936. This = reveals that the domains of several TPS family genes in XQC may have been lost in the evolution process after diverging from *A. thaliana*.

**Figure 6:**
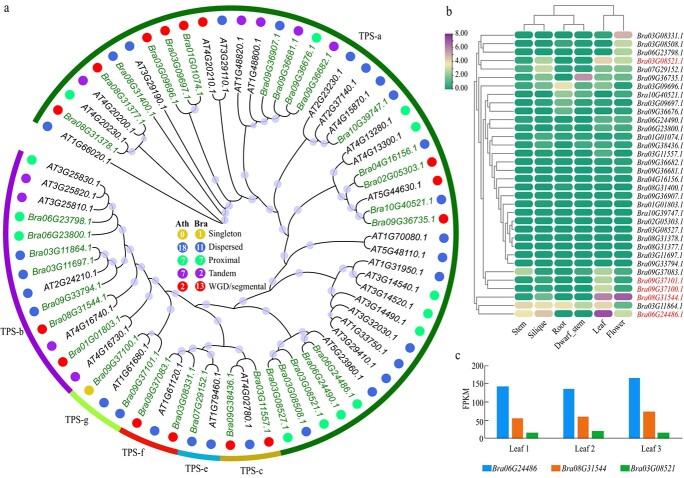
TPS gene family analysis. (**a**) Phylogenetic relationship of the TPS family genes in XQC and *Arabidopsis thaliana*. The phylogenetic tree was constructed using the maximum-likelihood model with 1000 bootstrap values. The light blue circle on the branch indicates that the bootstrap value was greater than 40%. The phylogenetic tree was divided in to six groups according to the classification of the *A. thaliana* TPS family genes. The different colored circles indicate the five duplication types. (**b**) The gene expression of the TPS gene family in six different tissues of XQC. The FPKM values of the gene expressions underwent a log2 transformation. (**c**) The gene expression of three up-regulated TPS family genes in the leaf with three replications.

Based on the classification of the TPS genes in *A. thaliana* [34], the TPS family genes were divided into six groups (Fig. 6a). The TPS-a group contains more genes than that of the TPS-e, TPS-g, TPS-c, and TPS-f groups. Furthermore, most TPS genes (18) in *A. thaliana* belonged to the dispersed duplication. However, the majority were linked to WGD/segmental duplication in XQC, including 13 TPS genes. For example, two TPS genes of XQC belonged to the WGD/segmental duplication in the TPS-c group, while only one gene was observed for this group in *A. thaliana.* The same phenomenon was also observed in the TPS-f group. These results demonstrate the important role played by WGD and segmental duplication in the TPS gene family of XQC.

The RNA-seq analysis reveals that most of the TPS family genes had low expression levels in six tissues of XQC (Fig. 6b). Four genes, *Bra06G24486.1*(TPS-a, FPKM: 147.55), *Bra03G08521.1* (TPS-a, FPKM: 17.06), *Bra09G37100.1* (TPS-g, FPKM: 6.45), and *Bra09G37101.1* (TPS-g, FPKM: 4.87), had higher expression levels in leaves compared to the other five tissues (Fig. 6b and c; Table S24, see online supplementary material). These genes may also play critical roles in the volatile aroma formation of XQC leaves. In addition, gene *Bra08G31544.1* (TPS-b) had higher expression levels in both flower (FPKM: 117.33) and leaf (FPKM: 62.44) compared to the other four tissues, indicating its potential role in the volatile aroma formation of the leaf and flower in XQC.

## Discussion

The XQC is a member of the *B. rapa*, which belongs to the Brassicaceae family. *B. rapa* has several subspecies, with the genomes of several subspecies released by previous literature, such as ssp. *pekinensis* (Chiifu) [11], ssp. *trilocularis* (yellow sarson) [36], and ssp. *chinensis* (Pakchoi) [8–10]. These released genomes provide rich resources for the genomic microevolution analysis of *B. rapa* in this study. As a ssp. *chinensis* of *B. rapa*, XQC has a strong fragrance, unlike other ssp. *chinensis*. To date, the genome of XQC is yet to be reported. Therefore, the released XQC genome provided in this study will provide rich resources for genomic studies of *B. rapa*.

Here, we reported a high-quality chromosomal-level XQC genome with the contig N50 of 15.15 Mb and scaffold N50 of 46.20 Mb. The values of these two main indicators were larger than the majority of previous studies on ssp. *chinensis* of *B. rapa* [8–10]. Furthermore, we determined for the first time that XQC has the closest relationship with *B. rapa* QGC and *B. rapa* Pakchoi via phylogenetic and syntenic analyses. Furthermore, we identified the occurrence of the latest WGT (21.59–24.40 Mya) event in XQC by correcting the evolution rate. Note that the majority of previous studies on *B. rapa* WGT time do not correct the evolutionary rate.

Previous research does not fully describe the molecular mechanism of fragrance and the related regulatory network of *B. rapa*. Here, based on the XQC genome, we detected 54 genes involved in the terpenoid biosynthesis, which take part in the formation of volatile fragrances in plants [37]. Furthermore, we identified 16 up-regulated genes in leaf rather than other tissues through transcriptome analysis, two of which were *PNO* genes (*Bra07G28783.1* and *Bra02G06135.1*) and one a *HDS* gene (*Bra10G40227.1*). These genes may play critical roles in the volatile aroma synthesis of XQC leaves. Finally, we identified 34 TPS family genes in XQC that play important roles in terpenoid biosynthesis. Our analysis also revealed WGD/segmental duplication to be key in the TPS gene family of XQC.

## Conclusion

In this study, we report and release the genome of XQC, which is of high quality. The total assembled length of the XQC genome is 466.11 Mb, and 47 570 genes were detected. A recent WGT event was identified in XQC after its divergence from *A. thaliana*, which was shared with other Brassica species. Some critical genes related to the terpenoid biosynthesis and TPS family genes were also detected in XQC. The XQC genomes, transcriptome, and the comparative analysis provide valuable resources for exploring the gene functions of XQC and other Brassicaceae species.

## Materials and methods

### Genome sequencing and estimation of genome size

Based on standard procedures, the DNA was obtained from XQC leaves by a QIAGEN kit (Shanghai, China). DNA quantification and purity were assessed using a Qubit® Fluorometer (Thermo Fisher Scientific, Waltham, MA, USA) and NanoDrop™ (NanoDrop Technologies, Wilmington, Delaware, USA) spectrophotometer (Nano Drop Technologies), respectively. Sequencing libraries were used to sequence the genome using the PacBio-Hifi (Pacific Biosciences, San Diego, CA, USA) and Illumina (Illumina, San Diego, CA, USA) platforms, referring to the previous studies [38, 39]. Finally, the genome assembly was performed using Hi-C technology. The genome size of XQC was estimated using the 17 nt k-mers based on the Illumina data [40].

### Quality control and genome assembly

The quality control of third-generation sequencing data was conducted using SMRT Link. Subreads were obtained after removing the adapter sequences and further filtered with a minimum length of 50. HiFi sequencing reads were obtained using CCS (min-rq = 0.99, min-passes = 3). The genome assembly of XQC was conducted with Hifiasm, which was developed according to the PacBio HiFi read feature [41].

### Assisted genome assembly and assessment by Hi-C

The quality control of the Hi-C sequencing data was implemented using HiCUP control and alignment control based on previous research [38, 42, 43]. The software ALLHiC was adopted to assist in the assembly of the XQC genome based on Hi-C technology [44]. The Juicebox program was used to visualize clustered bam files and XQC genomes [45]. Manual correction was performed based on the interaction strength of the chromosomes. The XQC genome was assessed using CEGMA and BUSCO [46, 47], while the alignment of second-generation reads to the XQC genome was performed with BWA [48].

### Genome annotation

The repeat sequences were detected based on *de novo* prediction and homologous alignment. First, RepeatModeler, RepeatScout [49], LTR_FINDER [50], and Piler [51] were used to construct a repeat sequence database for the *de novo* estimation with Repeatmasker. Repeatmasker and repeatproteinmask were then adopted to perform the alignment of homologous sequences by comparing with the RepBase [52, 53]. Simple sequence repeats were predicted based on previous research [54, 55]. miRNAs and snRNAs were predicted by INFERNAL [56], while rRNAs and tRNAs were predicted by BLAST and tRNAscan-SE, respectively [57]. Moreover, tandem repeat sequences were predicted by TRF [58].

### Gene prediction and functional annotation

SNAP [59], GlimmerHMM [60], and Augustus were used to conduct gene *de novo* predictions. Genewise and BLAST were employed for the gene homologous predictions [61, 62]. IntegrationModeler was adopted to combine the above results [63]. Finally, EVM gene prediction was corrected with RNA-seq data via the PASA [64]. The SwissProt, InterPro, KEGG, and TrEMBL databases were adopted to perform XQC gene annotation. TBtools was used to draw the repeat sequences, genes, and non-coding genes distribution on each XQC chromosome [65].

### Determination of gene families, phylogenetic relationship, and divergence time

Gene families were identified with OrthoFinder [66], while MCL was adopted to identify the multi- and single-copy gene families of XQC (-Inflation of 1.5). The contracted and expanded gene families of XQC and other examined species were performed with CAFE [67], and MUSCLE was adopted to conduct the multiple sequence alignments of single-copy family genes [68]. The phylogenetic tree was constructed using RAxML with the maximum-likelihood (ML) model [69]. Mcmctree of PAML was used to calculate the divergence time with the time correction points of the TimeTree website (http://timetree.org) [70, 71].

### RNA-seq analysis

The RNA was obtained from the root, stem, leaf, flower, silique, and dwarf stem of XQC. The NanoDrop spectrophotometer was adopted to assess the RNA purity. Agilent 2100 and Qubit were used to quantify the RNA integrity and concentration, respectively. The AMPure XP kit was used to construct the RNA-seq libraries. The sequencing of RNA was conducted using Illumina Hiseq 4000 (San Diego, CA, USA). The quality of RNA-seq data was assessed with FastQC. Bad quality reads and adaptors were removed using the trim-galore program. HISAT2 was adopted to map the clean reads to the XQC genome [72]. The expression value of each XQC gene was normalized to FPKM [73]. The DEGs were detected using DESeq (*P*-adj. < 0.05 and |log_2_(fold-change)| > 1) [74, 75].

### Genome collinearity analysis

Genome collinearity and dot plots were derived with WGDI (‘-icl’ model) [76, 77]. The length of the maximal collinearity gap was set to 50, and large gene families with more than 30 members were removed. We built collinear alignments for each examined species using the *A. thaliana* genome as the reference. The number of columns was assigned based on the WGD or WGT events of XQC and other examined species. Finally, syntenic alignments were drew by the Circos plot with the –ci module of WGDI [77]. MCscan toolkit in Python was adopted to draw the synteny of XQC and each examined species [78].

### Ks distribution and calculation

MUSCLE was employed to conduct sequence alignment [68]. The protein sequence alignment was converted into a codon alignment using PAL2NAL [79]. Finally, the Ka and Ks values were obtained using yn00 of PAML with the Nei-Gojobori method based on previous research [70, 80, 81]. WGDI was adopted to mark the Ks on the syntenic block with different colors [77]. The PeaksFit (−pf), Kspeaks (−kp), and KsFigures (−kf) tools of WGDI were used to illustrate the Ks density.

### Terpenoid biosynthesis pathway genes analysis

Terpenoid biosynthesis genes of XQC were predicted by comparisons with those of *A. thaliana* using Blastp (Identify>50%; E-value<1e-5; Score >200) according to previous research [36]. The TPS family genes were predicted with the Pfam accession numbers PF03936 and PF01397 (E-value <1e-5). Mafft was employed to perform protein sequence alignment [82]. FastTree was used to construct the phylogenetic tree with the JTT model (bootstrap set as 1000) [83, 84].

## Acknowledgements

This work was supported by the Suzhou Agricultural Science and Technology Innovation project (SNG2020065; SNG2020045), Suzhou Municipal Bureau of Agriculture and Rural Affairs, the National Natural Science Foundation of China (32172583), and the Natural Science Foundation of Hebei (C2021209005). The genome sequencing was performed in the Novogene Corporation.

## Author contributions

Z.L. was responsible for the project initiation. Z.L. and X.S. supervised and managed the project and research. Experiments and analyses were designed by Z.L., X.S., H.W., and Y.Z. Bioinformatic analyses were led by X.S., Z.L., Y.F., Y.Z., and S.S. The manuscript was written and revised by Z.L., X. S., Y.F., H.W., and Y.Z. All authors read and revised the manuscript.

## Data availability

The XQC genome sequence and RNA-seq datasets have been deposited in the Genome Sequence Archive [85] of the BIG Data Center [86], under accession numbers CRA010486 and CRA010488. They are publicly accessible at http://bigd.big.ac.cn/gsa. The genome sequences and annotation of XQC can be downloaded from the TBGR database (http://www.tbgr.org.cn) with the Genome ID of Pakchoi-XQC-v1.0 [30].

## Conflict of interest statement

The authors declare no competing interests.

## Supplementary data

Supplementary data is available at *Horticulture Research* online.

## Supplementary Material

Web_Material_uhad187
