# Supplementary material for: The high-quality sequencing of the Brassica rapa ‘XiangQingCai’ genome and exploration of genome evolution and genes related to volatile aroma
Source: Hortic Res. 2023 Sep 15;10(10):uhad187. doi: 10.1093/hr/uhad187 (PMC10611556; doi:10.1093/hr/uhad187)
Supplement: Web_Material_uhad187 [file web_material_uhad187.zip › Supplementary Figures 1-13.pdf]

Supplementary Figures 1-13

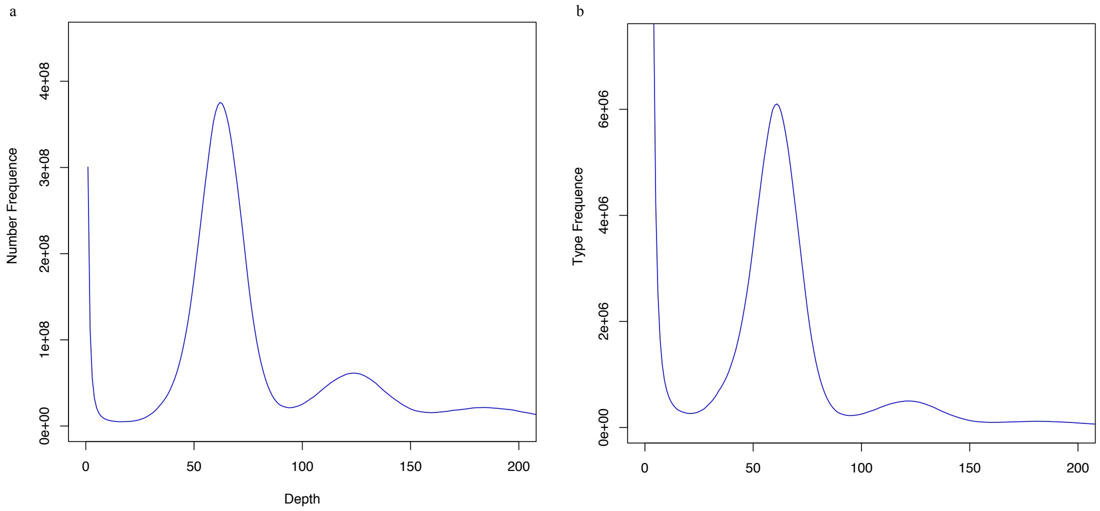

Supplementary Figure 1. K-mer distribution of the XQC genome. (a) K-mer=17 Depth and K-mer number frequency distribution. (b) K-mer=17 Depth and K-mer type frequency distribution.

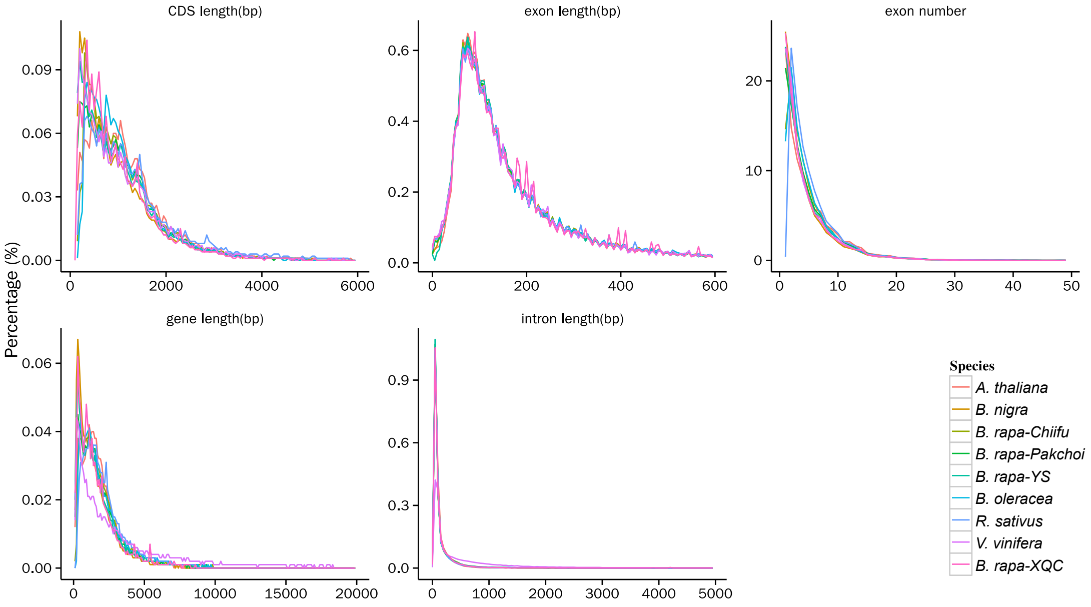

Supplementary Figure 2. Comparative analysis of CDS length, exon length, exon number, gene length, and intron length in XQC and other representative species.

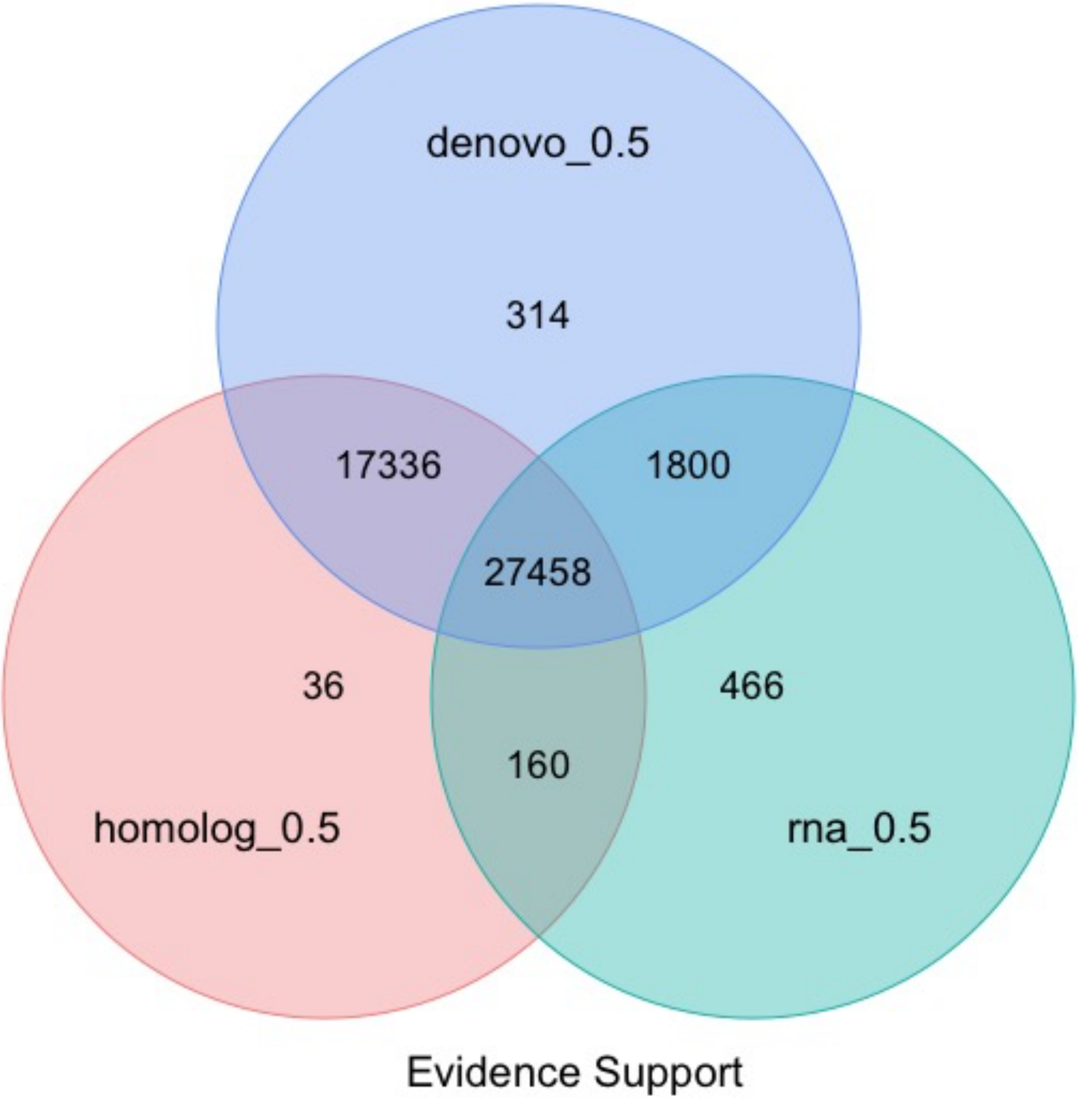

Supplementary Figure 3. The venn diagram of gene sets evidence in XQC genome.

De novo, EVM integrates genes supported by *Denovo* prediction; Homolog, genes supported by homologous prediction; RNA, genes supported by RNA-seq. The number indicates the gene number.

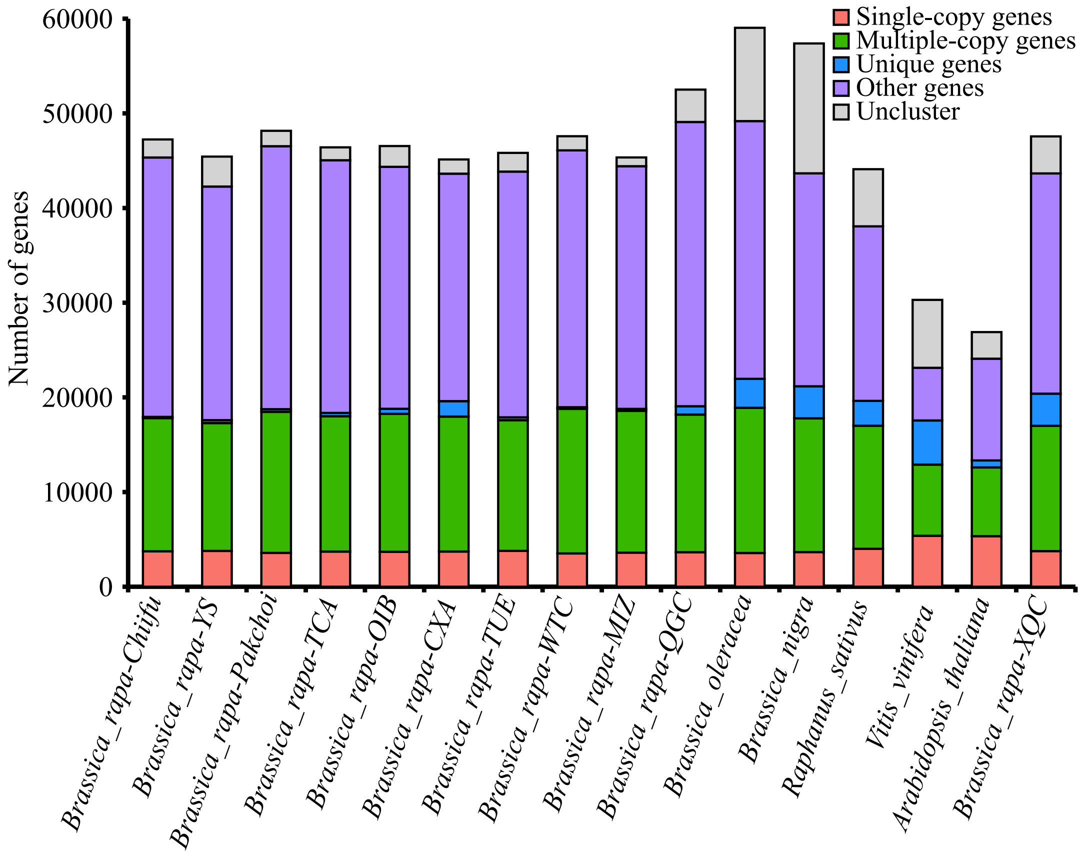

Supplementary Figure 4. Distribution of gene numbers and family sizes in XQC and 15 other representative species.

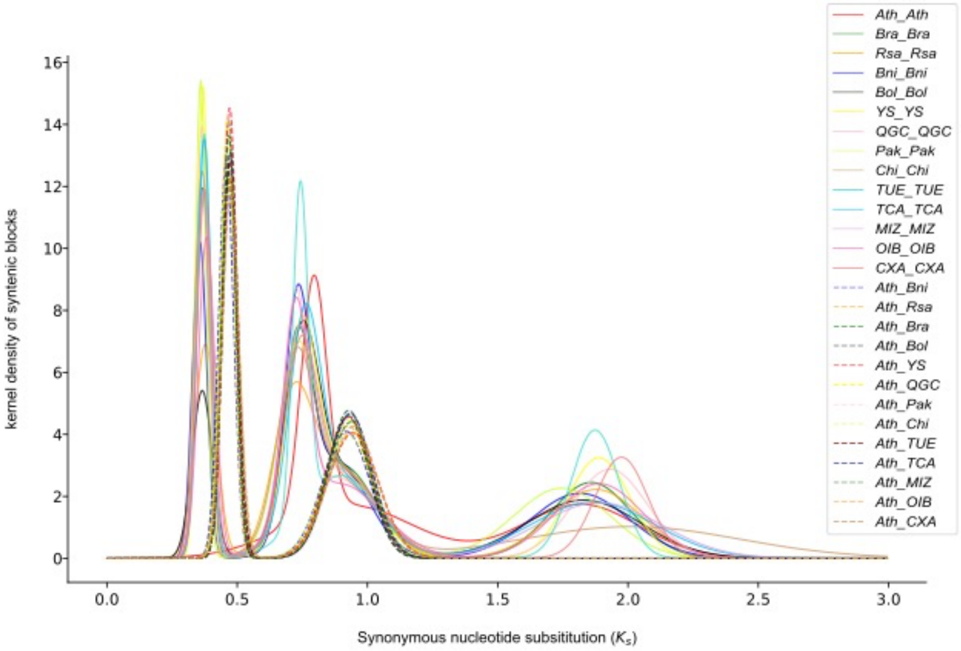

Supplementary Figure 5. The density of synonymous nucleotide substitutions per synonymous site (Ks) among collinear genes between XQC and other related species.

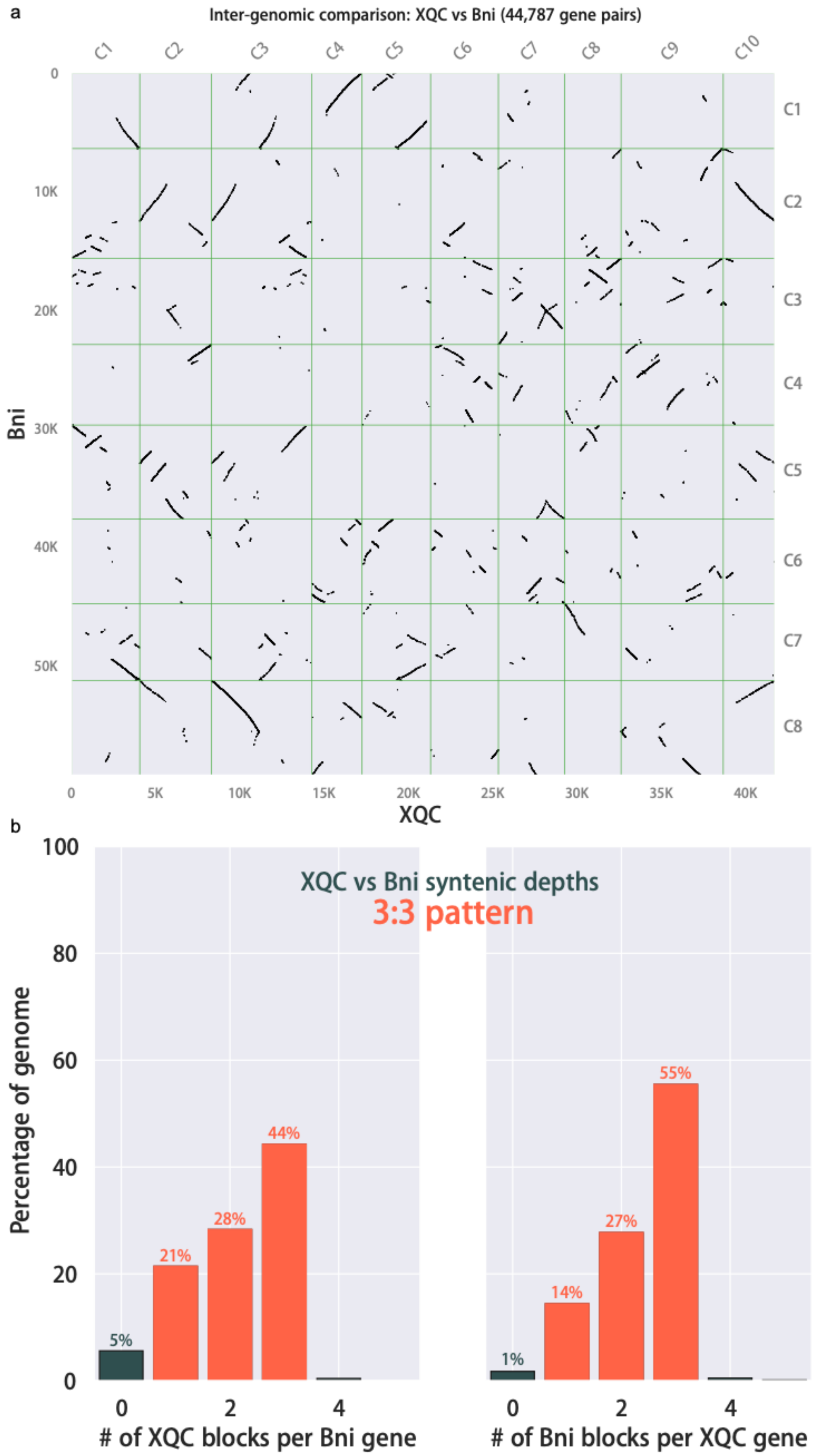

Supplementary Figure 6. The homologous dotplot and syntenic depth analysis between XQC and *B. nigra* (Bni) genome. (a) dotplot; (b) syntenic depths.

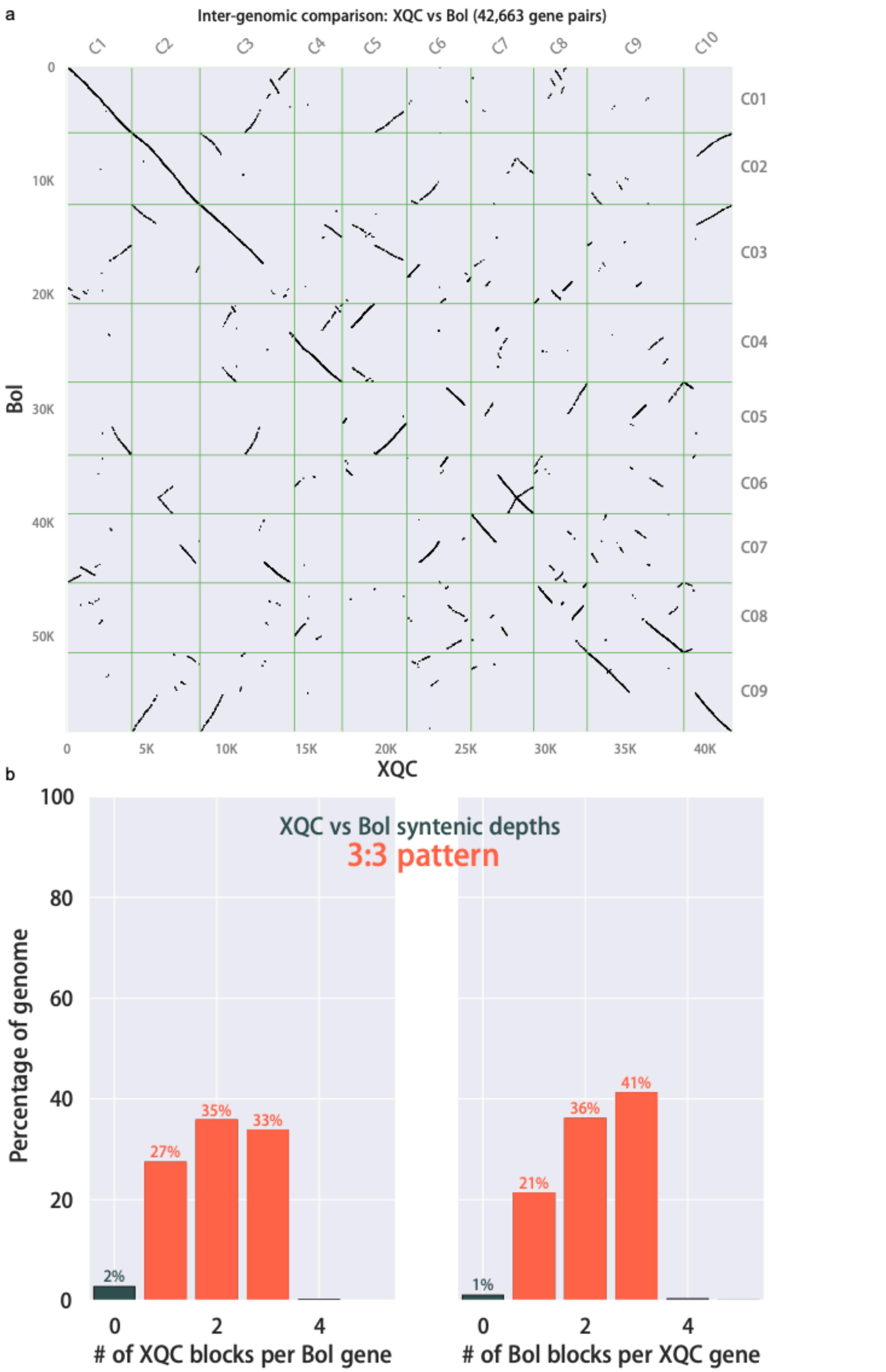

Supplementary Figure 7. The homologous dotplot and syntenic depth analysis between XQC and *B. oleracea* (Bol) genome. (a) dotplot; (b) syntenic depths.

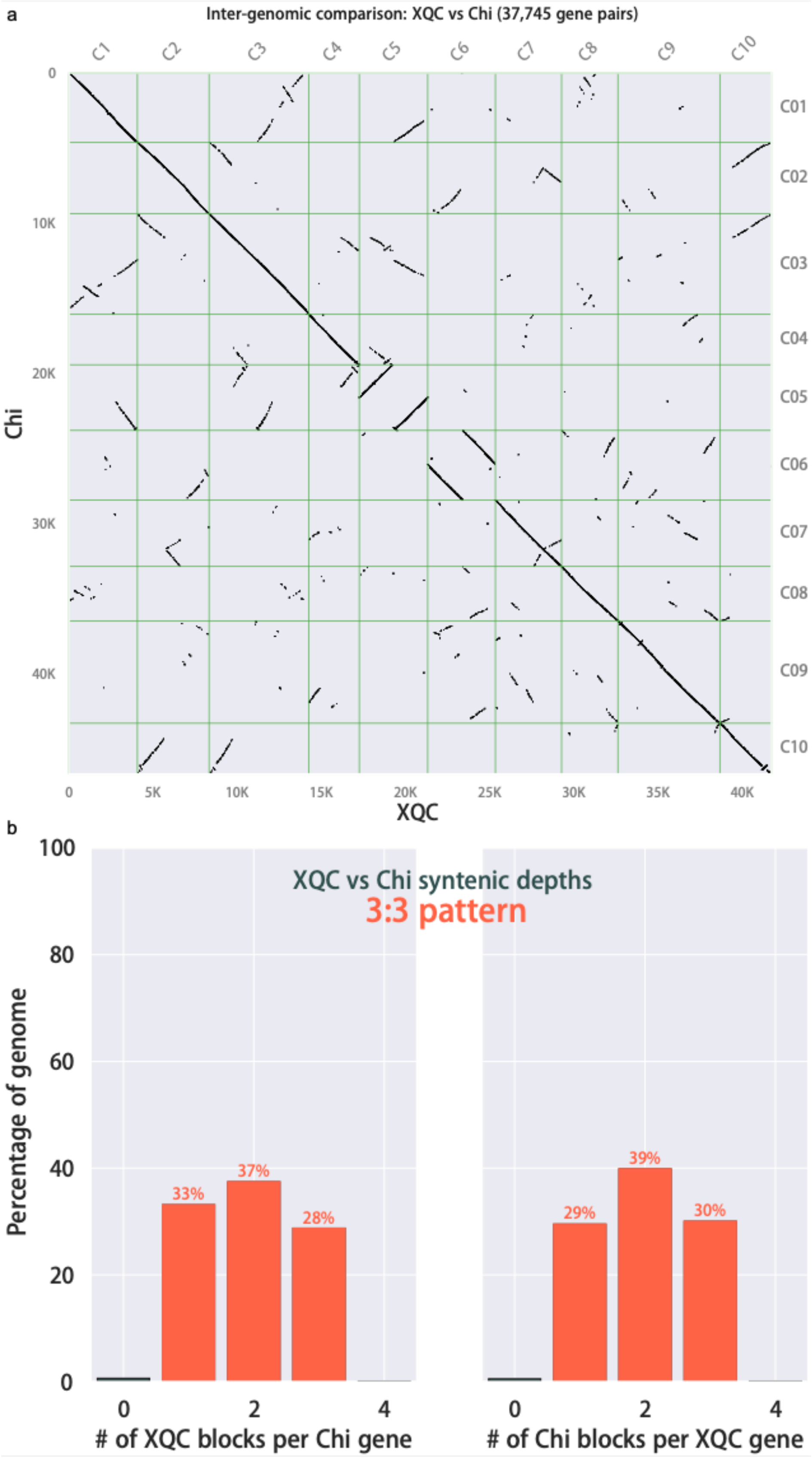

Supplementary Figure 8. The homologous dotplot and syntenic depth analysis between XQC and *B. rapa-Chiifu* (Chi) genome. (a) dotplot; (b) syntenic depths.

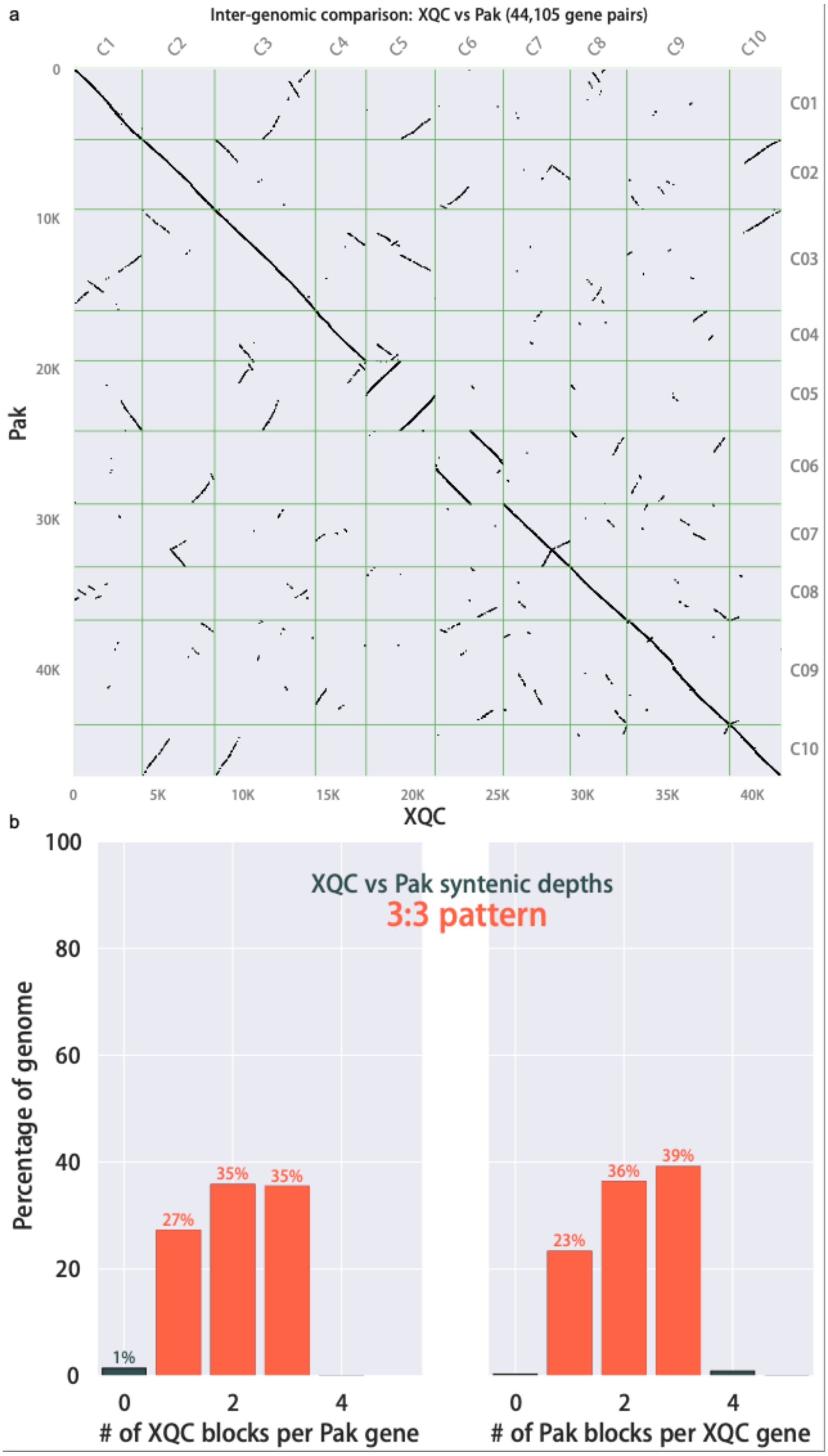

Supplementary Figure 9. The homologous dotplot and syntenic depth analysis between XQC and *B. rapa-Pakchoi* (Pak) genome. (a) dotplot; (b) syntenic depths.

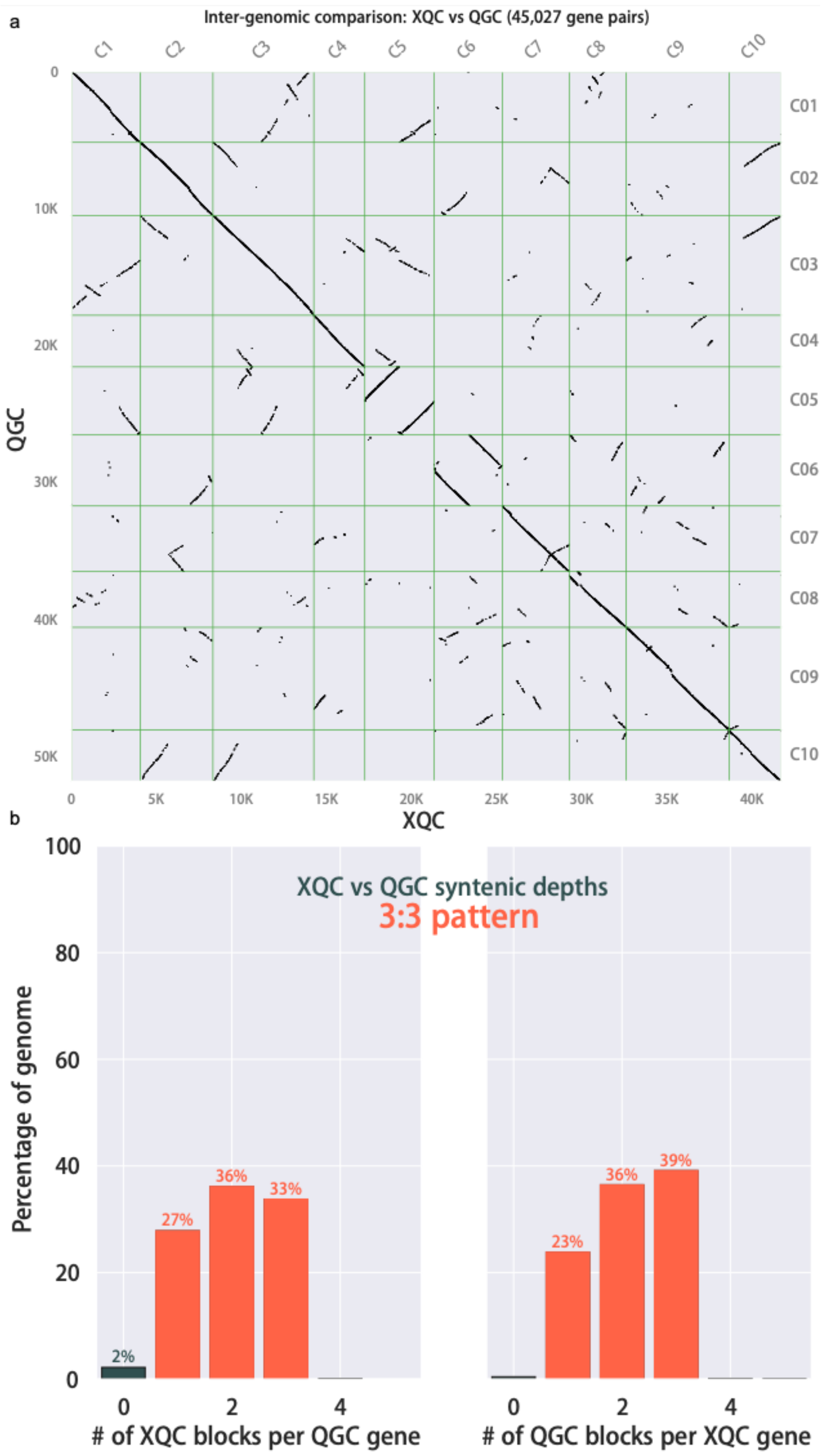

Supplementary Figure 10. The homologous dotplot and syntenic depth analysis between XQC and *B. rapa-QGC* (QGC) genome. (a) dotplot; (b) syntenic depths.

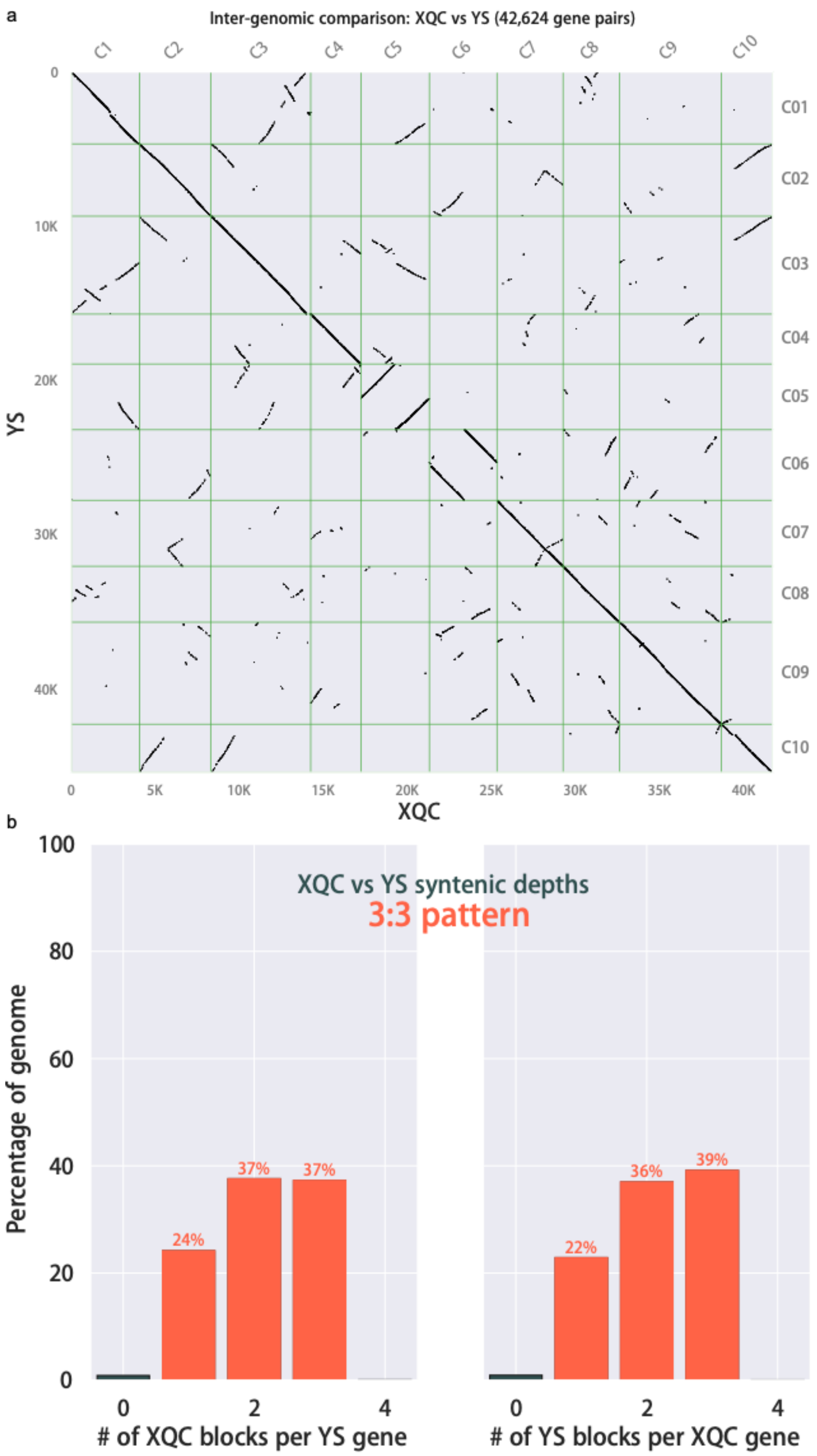

Supplementary Figure 11. The homologous dotplot and syntenic depth analysis between XQC and *B. rapa-YS* (YS) genome. (a) dotplot; (b) syntenic depths.

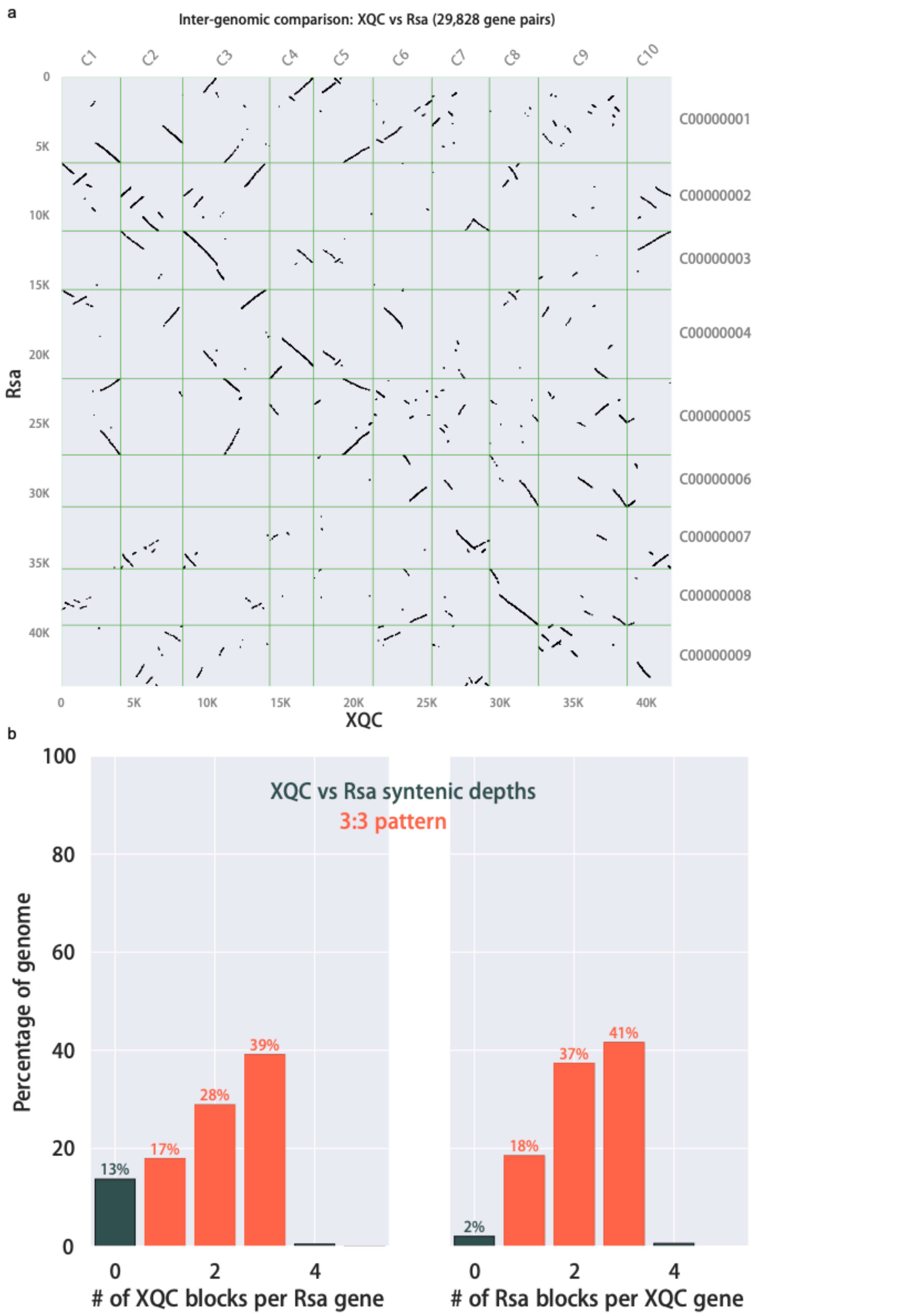

Supplementary Figure 12. The homologous dotplot and syntenic depth analysis between XQC and *R. sativus* (Rsa) genome. (a) dotplot; (b) syntenic depths.

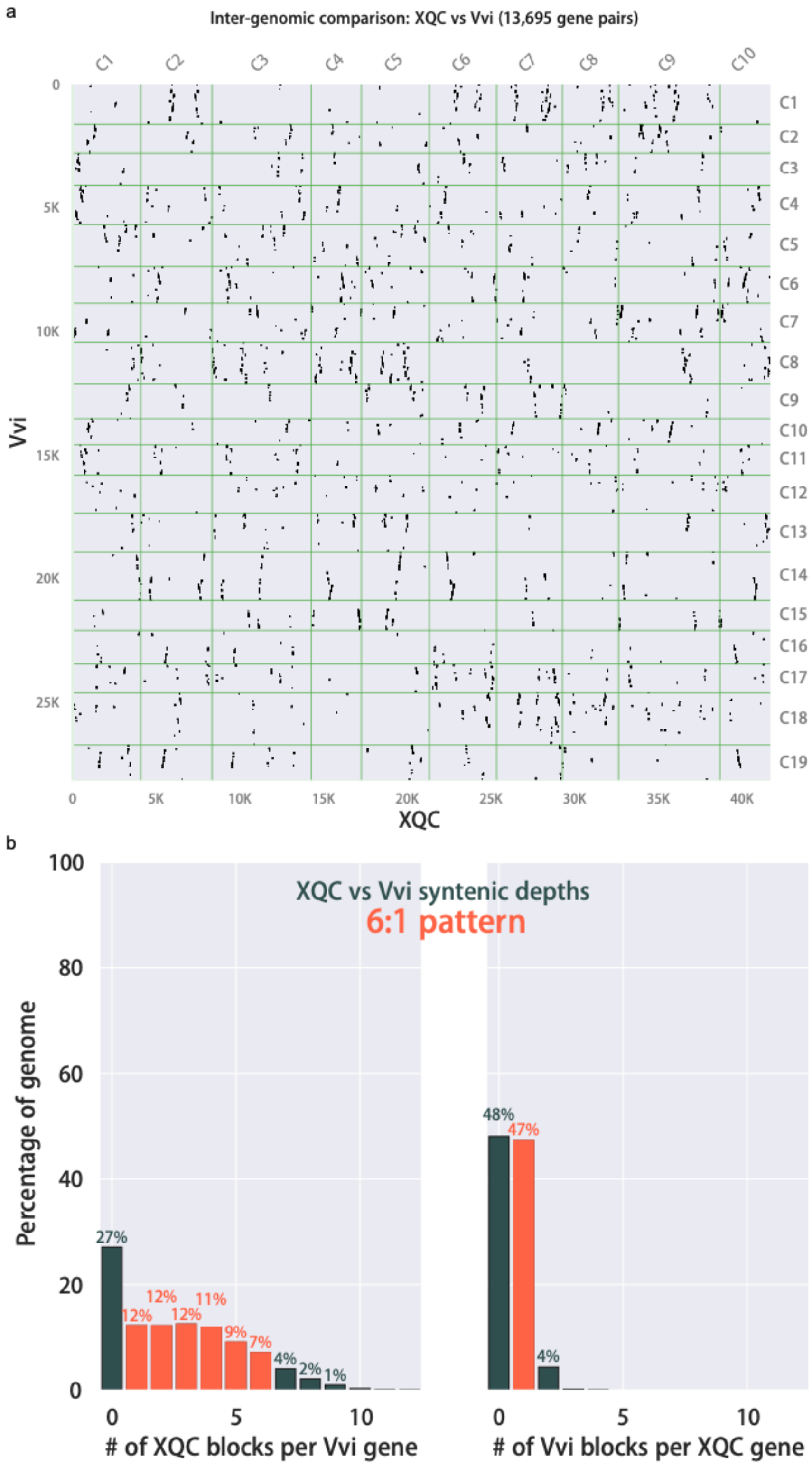

Supplementary Figure 13. The homologous dotplot and syntenic depth analysis between XQC and *V. vinifera* (Vvi) genome. (a) dotplot; (b) syntenic depths.
